# Elucidating the chemical and structural composition of breast cancer using Raman micro-spectroscopy

**DOI:** 10.17179/excli2021-3962

**Published:** 2021-07-02

**Authors:** Daniela Lazaro-Pacheco, Abeer M. Shaaban, Nicholas Akinwale Titiloye, Shazza Rehman, Ihtesham ur Rehman

**Affiliations:** 1 Department of Engineering, University of Exeter, Exeter, UK; 2 Department of Cellular Pathology, Queen Elizabeth Hospital Birmingham, and University of Birmingham, Birmingham, UK; 3 Department of Pathology, School of Medicine and Dentistry, Kwame Nkrumah University of Science & Technology, Kumasi, Ghana; 4 Department of Medical Oncology, Airedale NHS Foundation Trust, Airedale General Hospital, Steeton, West Yorkshire, UK; 5 Engineering Department, Faculty of Science and Technology, Lancaster University, Lancaster LA1 4YW, U.K.

**Keywords:** breast cancer, Raman spectroscopy, multivariate analysis

## Abstract

The current gold standard for breast cancer (BC) diagnosis is the histopathological assessment of biopsy samples. However, this approach limits the understanding of the disease in terms of biochemical changes. Raman spectroscopy has demonstrated its potential to provide diagnostic information and facilitate the prediction of the biochemical progression for different diseases in a rapid non-destructive manner. Raman micro-spectroscopy was used to characterize and differentiate breast cancer and normal breast samples. In this study, tissue microarrays of breast cancer biopsy samples (n=499) and normal breast (n=79) were analyzed using Raman micro-spectroscopy, and principal component analysis (PCA) was used for feature extraction. Linear discriminant analysis (LDA) was used for feature validation. Normal breast and breast cancer were successfully differentiated with a sensitivity of 90 % and specificity of 78 %. Dominance of lipids, specifically fatty acids, was identified in the normal tissue whereas proteins dominated the malignant spectra. Higher intensities of carotenoids, β-carotenoids, and cholesterol were identified in the normal breast while ceramide related peaks were mostly visible in the BC spectra. The biochemical characterization achieved with Raman micro-spectroscopy showed that this technique is a powerful and reliable tool for the monitoring and diagnosis of BC, regardless of the cohort heterogeneity. Raman spectroscopy also provided a powerful insight into the biochemical changes associated with the BC progression and evolution.

## Introduction

Breast cancer (BC) is the second most frequent cancer worldwide and the commonest cancer in women, according to the Global Cancer Statistics 2018 report (Bray et al., 2018[4]). With 630,000 estimated deaths in 2018, breast carcinomas had the first mortality rank among all types of cancers in females. The number of cases identified in 2040 is predicted to increase by a million, while mortality has been estimated to go up by 400,000 in comparison with the cases foreseen in 2020, according to the World Health Organization statistics (IARC, 2020[13]). Along with the increase in BC incidence, the shortage of trained pathologists has been estimated to worsen in the coming years. In the UK, a quarter of trained pathologists is less than ten years from retirement age. Moreover, only 3 % of the UK histopathological departments do currently have enough staff to meet clinical demand (The Royal College of Pathologists, 2018[37]).

An up-and-coming technology for the identification of BC and the assessment of cancerous sections is Raman spectroscopy. This vibrational spectroscopic technique focuses on the inelastic scattering of photons produced at relaxation after being excited with near-IR, visible or near-UV energy (Talari et al., 2015[33]). Raman spectroscopy allows identifying the chemical bond and functional groups within the tissue, facilitating its chemical characterization (Rehman, 2013[28]). The identification of chemical changes at the tissue level can be correlated with the presence of disease even before morphological changes take place (Brozek-Pluska et al., 2018[5]). Furthermore, Raman spectroscopy allows the label-free analysis of mounted tissue, and with the coupling of confocal microscopy and mechanical controlled stages, it can facilitate high-throughput analysis of samples (Lazaro-Pacheco et al., 2020[20]). The combination of other sampling techniques such as tissue microarrays (TMA) can reduce the amount of tissue needed for assessment while allowing a cost-effective, high throughput analysis (Camp et al., 2000[7]). The combination of both technologies, Raman spectroscopy and TMAs, could not only help to alleviate the pressure in histopathological department assisting with the identification of BC but also help to understand the underlying biochemistry associated with the progression and evolution of the disease as well as early detection (Keller et al., 2006[15]).

The use of Raman spectroscopy has allowed the identification of biological components within a variety of tissue in addition to their quantities, quality and structure (Talari et al., 2019[36]). The classification of different breast cancer and normal breast cell lines representative of different receptor status has been successfully classified with a 100 % sensitivity and 91 % specificity (Talari et al., 2015[34]). The classifications achieved when combining Raman spectroscopy and multivariate analysis have enabled differentiating between normal and malignant tissue using basic differences in the concentration of biochemical compounds within the cells. Validating the technique in normal mammary tissue and patient tumor samples would therefore be an important step towards understanding the chemical changes associated with mammary tumorigenesis. 

In this study, African breast cancer samples assembled in TMAs were analyzed using Raman micro-spectroscopy. Principal component analysis (PCA) was used to identify the biochemical changes and spectral biomarkers associated with the presence of cancer, and linear discriminant analysis (LDA) was used to validate the findings. 

## Materials and Methods

### Sample information and preparation

The study investigated cancer samples and normal breast tissue arranged in seven different TMAs. The cancerous samples corresponded to breast samples from Nigerian patients, and the normal breast tissue was obtained from surgical breast reductions. The cohort in use was arranged into TMAs under the Ethical approval REC No. 06/Q1206/180. The tissue cores were 5 μm thick and 0.6 mm in diameter. To help with the orientation, different human and bovine tissues were added to the TMAs. The TMAs were prepared as reported by Pinder et al., (2013[26]) and Titiloye et al. (2016[39]). 

Six TMAs containing breast cancer samples (n=378) and one TMA composed of normal breast (n=134) were received in duplicates. Continuous cuts of the same breast section were placed on each duplicate slide. From each duplicate set, one slide was used for Haematoxylin and Eosin (H&E) staining and the other for spectroscopic analysis after dewaxing. H&E staining and assessment was done to confirm the areas of interest such as cancerous and normal epithelial tissue. Mian's dewaxing protocol (Mian et al., 2014[24]) was modified by reducing the time in xylene to 10 minutes, considering the sample thickness.

### Spectra acquisition 

Some loss of TMA cores and/or folding of sections, a well recognized issue associated with TMA sectioning and preparation, was observed. The eligibility criteria for Raman analysis required the availability of both corresponding H&E and its dewaxed tissue core and in the cancerous TMA´s, identifiable cancerous areas by H&E staining. 

Twenty Raman spectra were collected from the epithelial tissue of each sample, either cancerous or healthy. The Raman spectral data was collected using a Raman microscope DXR™ (Thermo Scientific, Waltham, MA, USA) using a 50X long work distance objective, a 532 nm diode laser with a 10 mW laser power and a 50 μm pinhole within 3500-400 cm^-1^ spectral range.

### Pre-processing and analysis

Fluorescence can be present in biological samples, therefore, all the spectral data was fluorescence corrected using a six polynomial algorithm, by employing Omnic™ (Thermo Scientific, Waltham, MA, USA) software. Twenty spectra per sample were averaged and used for multivariate analysis. The Unscrambler X 10.2™ software (Camo software, Oslo, Norway) was used for statistical analysis and pre-processing. The spectral data was pre-processed using baseline, Savitzky-Golay smoothing and standard normal variate (SNV) corrections. Principal component analysis (PCA) was performed on the following spectral regions: fingerprint 1800-500 cm^-1^, amides 1800-1140 cm^-1^, amino acids and nucleic acids 980-600 cm^-1^, lipids 3100-2680 cm^-1^, and hydroxyproline /proline 960-810 cm^-1^. PCA was followed by linear discriminant analysis (LDA) for validation. LDA was performed leaving out five samples from each group at each pass until the total number of spectra of each type were predicted. The sensitivity and specificity of the model were calculated using the correctly classified false positive, false negative and true negative.

## Results and Discussion

Based on the inclusion criteria, a total of 578 samples were analyzed with Raman microspectroscopy comprising 499 breast cancer tumors and 79 normal breast sections. The cancerous TMAs were received with the following anonymised information: histological tumor type, tumor grade, estrogen receptor (ER), progesterone receptor (PR) and HER2 status, and patient sex and age at diagnosis. Based on the molecular profile, the invasive carcinomas were classified into luminal, her2 positive or triple negative (TNBC). The clinicopathological features of the cancerous and normal cohort are presented in Table 1(Tab. 1). Ductal NST (no-special-type) carcinoma was the dominant histological subtype accounting for 87.3 % of the cancerous cases. Ductal carcinoma in situ (DCIS) was also included in the analysis.

**Figure 1:**
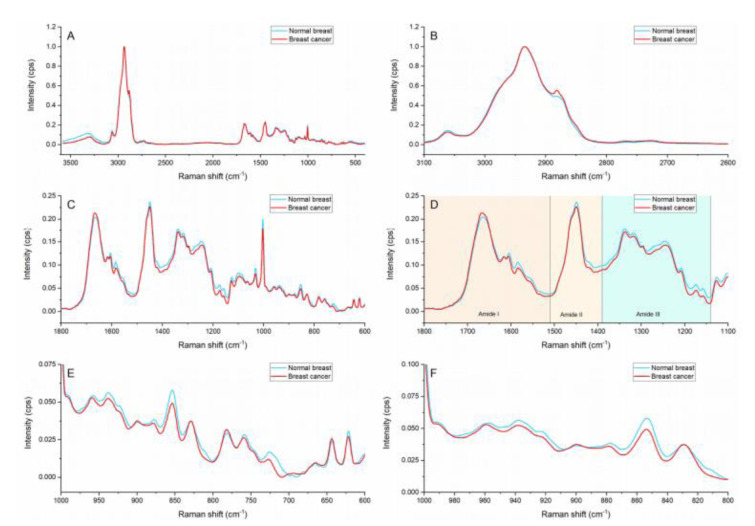
Raman spectra of cancerous breast and normal breast. (A) General region (3600-400 cm^-1^), (B) Lipid region (3,100-2,680 cm^-1^), (C) Fingerprint region (1,800-500 cm^-1^), (D) Amides region (1,800-1,140 cm^-1^) indicating: Amide I (1,800-1,510 cm^-1^), Amide II (1,510-1,390 cm^-1^) and Amide III (1,390-1,140 cm^-1^) regions. (E) Amino acid and nucleic acids regions (980-600 cm-1), and (F) Hydroxyproline and proline region (810-960 cm^-1^).

The average spectra of the cancerous epithelia and the normal epithelia within the breast samples are presented in Figure 1(Fig. 1). Breast cancer and normal breast spectral profiles showed subtle differences in intensity and peak position. The spectral profile of breast cancer showed higher intensity on the lipid area (Figure 1B(Fig. 1)) and the peaks located at ≈1667 and ≈1295 cm^-1^. Noticeable changes in peak ratios and peak shapes can be observed in the amide region (Figure 1D(Fig. 1)). Furthermore, subtle peak shifts are present on the amino and amino acid region (Figure 1E(Fig. 1)).

The peak located at ≈1295 cm^-1^ is only visible in the breast cancer spectral profile. This peak is representative of ceramides which form part of the structural components of sphingolipids and are considered vital biomolecules in the carcinogenesis process. Sphingolipids are membrane lipids and work as signaling molecules that are involved in cell growth and differentiation, apoptosis, senescence and proliferation (Hartmann et al., 2012[12]). Sphingolipids regulate various cellular processes linked to cancer development, progression, metastasis and therapy resistance, identification. Ceramide levels are statistically significantly increased (12-fold higher) in breast cancer tissue in comparison with benign tumors and normal breast (Schiffmann et al., 2009[32]). The identification of ceramides via Raman spectroscopy at tissue can therefore be suggested as a robust biomarker for breast cancer.

The I_1667_/I_1449_ ratio compares the protein to lipid content being represented by the C=C stretch in amide I and the CH_2_ scissoring in lipids. The calculated ratios showed that the normal breast had a lower protein to lipid proportion in comparison with the breast cancer tissue (0.86 vs 0.93).

### Multivariate analysis

Comparison of breast cancer and normal breast tissue in different spectral regions were performed using PCA. Different scores and loadings are produced as output of this analysis. Both are complementary to each other to interpret the data. The scores show the dispersion of the samples based on the variance along the analyzed area. The loadings are a representation of the contribution of the Raman bands. In the loading for a particular principal component (PC), it can be assumed that the higher the intensity, the higher the contribution of that specific peak for the classification. The PCA model created showed a sensitivity of 90 % and a specificity of 78 %.

#### General region (4000-400 cm^-1^)

**Figure 2:**
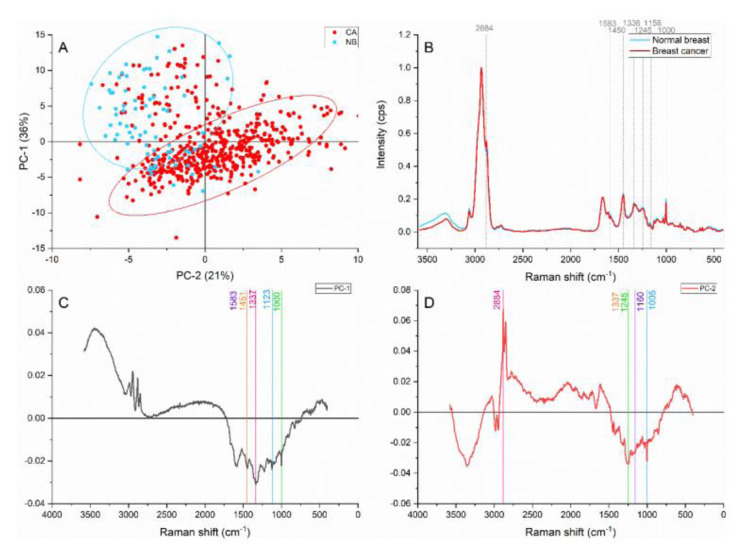
Principal component analysis on the general region (4,000-400 cm^-1^). (A) Score plot using PC-1 and PC-2 accounting for 57 % of the variance-ellipses were drawn subjectively based on visual trends, (B) Average Raman spectral profile for breast cancer and normal breast. Dotted lines represent the peaks found as responsible for the separation based on the loadings presented in C&D, (C) PC-1 loading, (D) PC-2 loading

When PCA was applied to the general spectral region, a clear separation and grouping were achieved with the PC-1 and PC-2 accounting for 57 % of the variance (Figure 2(Fig. 2)). The peaks located at 2884, 1583, 1450, 1336, 1245, 1159, 1122, and 1004 cm^-1^ were identified as responsible for the grouping and separation.

The peak located at ≈2884 cm^-1^ attributed to the CH_2_ and CH stretching of lipids and proteins (Talari et al., 2015[33]) contributed more in the cancer area than in the normal breast. Tryptophan and the C=C bending mode of phenylalanine (Lau et al., 2003[18]) identified by the ≈1583 cm^-1^ band also showed greater intensities on the cancer samples. The content differences represented by these two peaks might be caused by the accelerated and active cell component production characteristic of cancer development and progression as well as the structural components in highly dense samples. Furthermore, the cancerous band located at ≈1450 cm^-1^ presented higher intensities than the normal counterpart. This band has been reported as a CH_2_ bending characteristic of malignant tissues (Talari et al., 2015[33]).

The ≈1336 cm^-1^ peak has been assigned to different components, such as the amide III, and polynucleotide chains, and CH_2_ wagging vibrations from glycine backbone and proline side chain in collagen (Lazaro-Pacheco et al., 2020[20]). Breast cancer samples presented higher intensities in this and which suggests higher content of proteins, nucleic acids, and collagen in the malignant samples. The amide III contribution in breast cancer was confirmed by the ≈1245 cm^-1^ peak, which was identified by the PC-2.

The normal breast samples presented higher intensities than the average on the 1159 cm^-1^ peak associated with the C=C stretch mode characteristic of carotenoids and β-carotenoids. Besides, the band located at 1122 cm^-1^ representing C-C stretch on carotene and breast lipids also presented a similar behavior. Carotenoids offer oxidative damage protection, upregulate connexion junctional communication and prevent the lipid oxidation. Moreover, normal breast tissue is known to possess a higher quantity of carotenoids than cancerous tissue, acting as a fatty acids reservoir (Abramczyk et al., 2012[2]). The decreased intensity of carotenoids contributions suggested a lower content of these components in the malignant tissue. Brożek-Płuska et al. have suggested that the oxidation of aging-related pigments and the peroxidation of lipids are responsible for the decline of carotenoids in cancerous breast (Brożek-Płuska et al., 2008[6]).

Furthermore, the phenylalanine peak found at 1000-1004 cm^-1^ (Talari et al., 2015[33]) presented greater contributions in cancerous samples. This peak could be associated with stromal components and with structural features of the epithelial cells. Collagen fibre deposition, rearrangement of fibres and overproduction occurring when tumors develop and grow have been reported (Conklin et al., 2011[9]), and it could be expected in our samples. However, as the epithelial tissue was targeted on this study, the assignment is representative of higher cell density as a result of abnormal proliferation. 

#### Fingerprint region (1800-500 cm^-1^)

**Figure 3:**
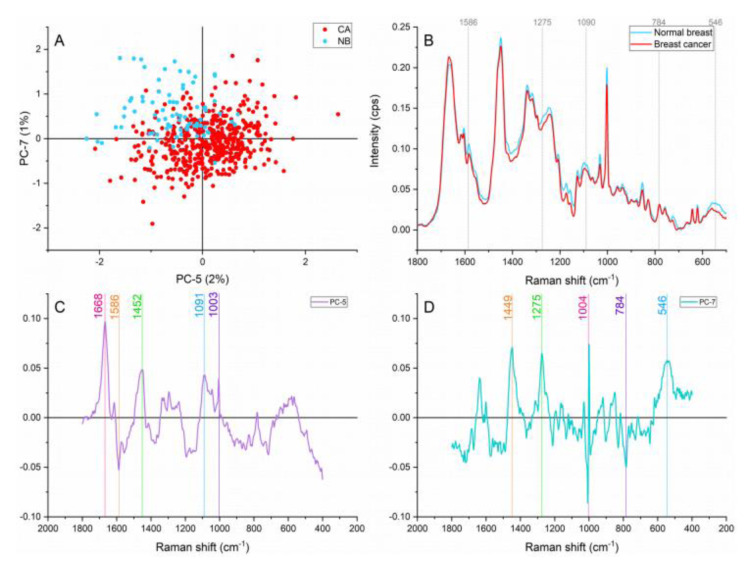
Principal component analysis on the fingerprint region (1,800-500 cm^-1^). (A) Score plot using PC-5 and PC-7 accounting for 3 % of the variance, (B) Average Raman spectral profile for breast cancer and normal breast. Dotted lines represent the peaks found as responsible for the separation based on the loadings presented in C&D, (C) PC-5 loading, (D) PC-7 loading

Narrowing down the analysis to the fingerprint region revealed 1275, 1090, 784, and 546 cm^-1^ as new peaks responsible for the separation and grouping of samples as shown in Figure 3(Fig. 3). Representing nucleic acids bands, the peaks located at ≈1090 and 784 cm^-1^ are assigned to the symmetric phosphate stretching vibrations and ring breathing modes in DNA and RNA bases (Rehman, 2013[28]; Yu et al., 2006[41]). These two bands showed higher intensities in the cancer samples signifying higher cell density and genetic material overproduction. This overproducing behaviour is characteristic of early and late stages of carcinogenesis. Besides, the phosphate assignment can also serve as an indicator of higher metabolic activity (Talari et al., 2015[34]).

The band found at ≈1275 cm^-1^ assigned to amide III contributions is a protein characteristic vibration but is also considers a collagen specific assignment (Talari et al., 2015[33]). Higher contributions of this collagen band were identified in the normal breast in comparison with the cancerous breast. Amide III represents α helix conformations (Chen and Lord, 1974[8]). The lower intensity in α helix contributions in the cancerous breast suggested conformational changes in the cancerous tissue. This finding is in agreement with our FTIR results (Lazaro-Pacheco et al., 2020[19]).

A cholesterol related peak located at ≈546 cm^-1^ presented greater contributions in the normal breast. Starting stable cholesterol content is expected in normal breast (Kneipp et al., 2003[16]). However, oxidized and modified cholesterol has been reported as a sign of histological progression from normal epithelium to hyperplasia and could explain the low contribution of this component in the malignant tissue (Wrensch et al., 1989[40]). 

#### Amide region (1800-1140 cm^-1^)

**Figure 4:**
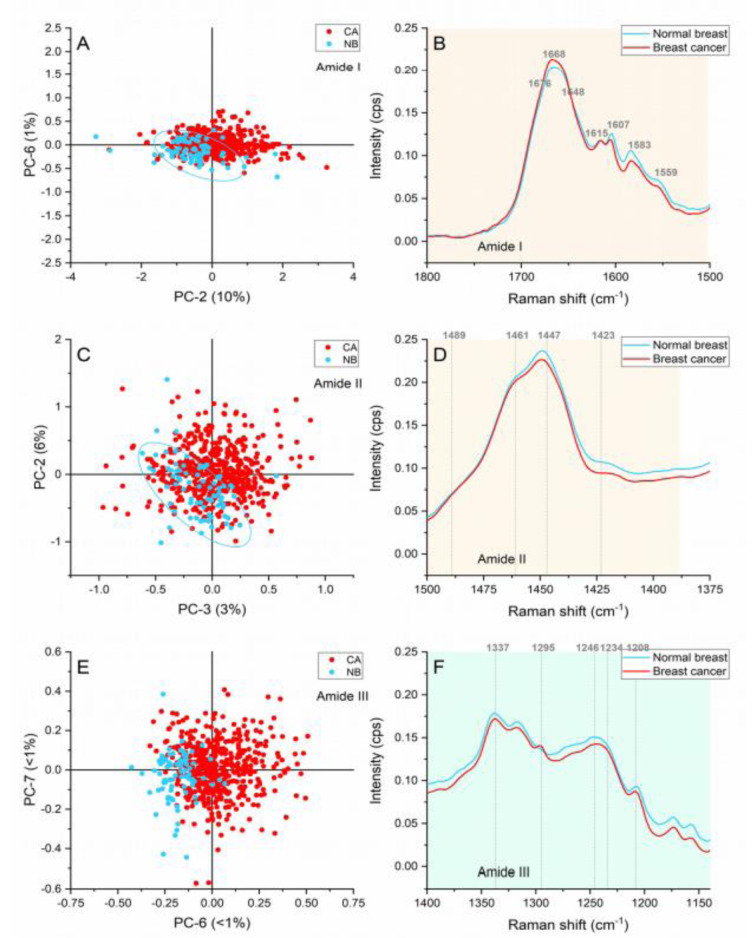
Principal component analysis of the amide region. (A) Score plot of the amide I region (1,800-1,510 cm^-1^), (B) Average Raman spectral profile for breast cancer and normal breast with peaks responsible for the separation in the amide I region, (C) Score plot of the amide II region (1,510-1,390 cm^-1^), (D) Average Raman spectral profile for breast cancer and normal breast with peaks responsible for the separation in the amide II region, (E) Score plot of the amide III region (1,390-1,140 cm^-1^), (F) Average Raman spectra profile for breast cancer and normal breast with peaks responsible for the separation in the amide III region. Ellipses were drawn subjectively based on visual trends.

The bands found to contribute towards the grouping of samples when the amide region was analyzed are presented in Figure 4(Fig. 4). 

#### Amide I region (1,800- 1,510 cm^-1^)

The first peak located at ≈1667 cm^-1^ is caused by the carbonyl (C=O) stretching vibrations of the amide I, but it is also representative of the α-helix structures of proteins (Rehman et al., 2007[29]). The spectral profiles showed an upshift from 1663 cm^-1^ in the normal breast to 1667 cm^1^ in the cancerous area as shown in Figure 4B(Fig. 4). Manoharan has previously reported this shift towards higher wavenumbers in addition to a widening of the peak (Manoharan et al., 1998[21]). In our case, a minimal widening can be appreciated in the breast cancer spectral profile, but a higher intensity has been confirmed in the score plot. The higher intensities in the malignant samples suggest a greater contribution of proteins with dominant α-helix structure. 

The peak located at ≈1648 cm^-1^ related to random coil vibrations presented higher intensities in comparison with breast cancer samples. In addition, the ≈1676 cm^-1^ peak assigned to amide I β-sheet conformations showed higher intensities in the cancerous spectra. These conformational changes are caused by ECM adaptation in hypoxia (Talari et al., 2017[35]) and have been differentiators of normal and cancerous cells in *in vitro* studies (Talari et al., 2015[34]).

The peaks located at ≈1615 cm^-1^ and 1605 cm^-1^) representing tyrosine phenylalanine, tryptophan, and C=C vibrations in proteins (Rehman, 2013[28]) had higher intensities in the cancerous area of the spectra. These responses serve as indications of overproduction of cellular components leading to abnormal proliferation.

#### Amide II region (1,510-1,390 cm^-1^)

The bands located at ≈1461 and 1489 cm^-1^ presented greater intensities in the cancerous samples. These bands represent deoxyribose vibrations and DNA vibrations. The increased intensities in the cancerous samples suggest an accelerated genetic material production leading to cell division and aberrant behavior. Similarly, variable intensities in the CH deformation-representative band located at ≈1448, suggested changes linked to protein and lipid content.

Normal breast samples presented higher intensities in the 1423 cm^-1^ band associated with the N-H in plane deformation in proteins (Talari et al., 2015[33]). This freedom for deformation might be caused by the vibrational modes found in strict intensely packed structures in normal tissue in comparison with the much-relaxed β-structures in malignant tissue (Rygula et al., 2013[31]).

#### Amide III region (1,390-1,140 cm^-1^)

Ceramides (≈1295 cm^-1^), amide III from a concerted ring mode(≈1234 cm^-1 ^), and tryptophan, phenylalanine, and amide III vibrations characteristic of proteins (≈1208 cm^-1^) were identified as responsible of the grouping of the cancerous and normal breast. These peaks presented larger intensities in the cancerous areas, which suggest higher metabolic activity and cell density in the malignant tissue. 

Nucleic acids variation represented by the ≈1291 cm^-1^ peak linked to cytosine vibrations (Rehman, 2013[28]) were confirmed for the cancerous areas. The production of genetic material and cellular content is expected as part of the aberrant proliferation in malignancies. 

The bands found at ≈1243 and ≈1204 cm^-1^ are considered collagen assignments. The CH_2 _wagging of the glycine backbone and proline side changes contribute considerably to the total intensity of this peak (Talari et al., 2015[33]). Normal breast presented higher contributions of these bands suggesting the inclusion of collagen in the Raman data collection. This inclusion has been considered due to the lower cell density in the normal epithelia. Nevertheless, some cancerous samples presented considerable contributions to these two peaks. These contributions in the cancer tissue could represent the extracellular matrix preparation to facilitate further invasion and hypoxia survival (Kumar et al., 2013[17]; Talari et al., 2017[35]). Among the adaptation changes, the overproduction of collagen is present by fibroblast stimulation (Provenzano et al., 2008[27]).

#### Aminoacid and nucleic acid region (980-600 cm^-1^)

**Figure 5:**
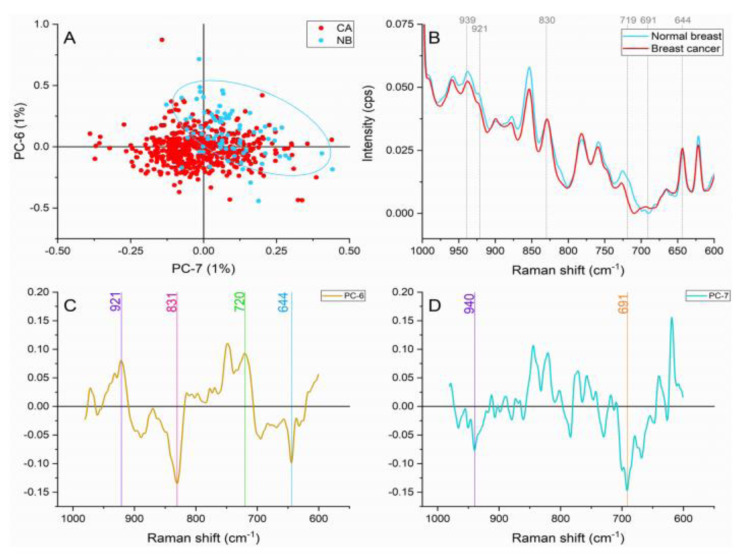
Principal component analysis on amino acids and nucleic acids region (980-600 cm^-1^). (A) Score plot using PC-6 and PC-7 accounting for 2 % of the variance-ellipses were drawn subjectively based on visual trends, (B) Average Raman spectral profile for breast cancer and normal breast. Dotted lines represent the peaks found as responsible for the separation based on the loadings presented in C&D, (C) PC-6 loading, (D) PC-7 loading

The band at ≈921 cm^1^ caused by the C-C stretch of proline ring, and the peak found at ≈939 cm^-1^ represent the C-C stretch of the skeletal collagen backbone (proline and hydroxyproline) presented important contributions in both tissue types as shown in Figure 5(Fig. 5). The collagen contributions can be explaining with the stromal inclusion in the normal breast samples and the overproduction and hyperactive activity of carcinoma-associated fibroblasts (CAFs) in the malignant tissue (Orimo et al., 2005[25]).

Breast cancer samples presented higher intensities in comparison with normal breast spectra on the peaks located at ≈830 cm^-1^ produced by PO_2_^-^ stretching of nucleic acids, ≈644 cm^-1^ representing C-C twisting mode of tyrosine and phenylalanine characteristic of proteins, and ≈691 cm^-1^ caused by cholesterol ester vibrations. The increased intensities in the malignant tissue are caused by a higher metabolic activity as reported in previous works (Rehman et al., 2007[29]; Talari et al., 2015[34]).

Normal breast spectra presented greater intensities in the ≈719 cm^-1^ peak assigned to the C-N vibration in the membrane phospholipid head (Talari et al., 2015[33]). A number of authors have proposed a decrease in phospholipids in malignant breast tissue (Glunde et al., 2004[11]; Kast et al., 2008[14]; Ruiz-Cabello and Cohen, 1992[30]; Ting et al., 1996[38]). As a cause of this reduction, the synthesis and breakdown of choline phospholipids caused by specific genetic and phospholipids alterations affecting the membrane fluidity have been proposed. 

#### Lipid region (3100-2680 cm^-1^)

**Figure 6:**
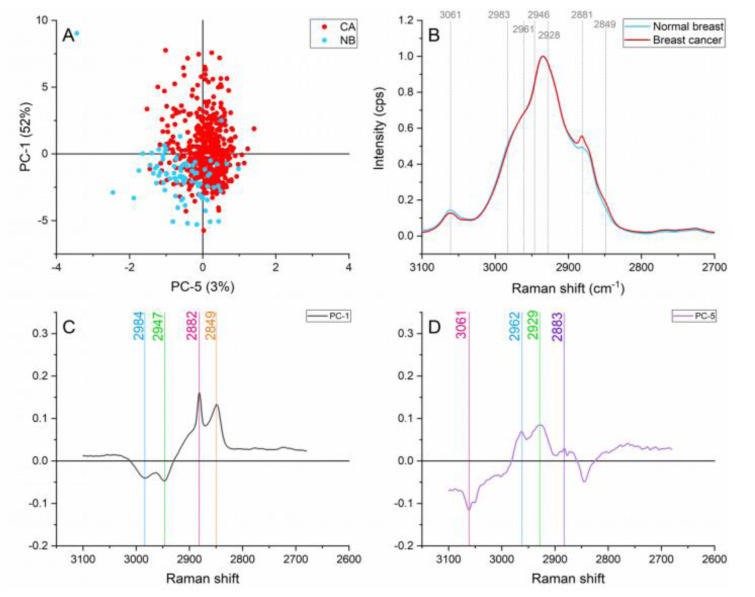
Principal component analysis on the lipid region (3,100-2,680 cm^-1^). (A) Score plot using PC-1 and PC-5 accounting for 55 % of the variance, (B) Average Raman spectral profile for breast cancer and normal breast. Dotted lines represent the peaks found as responsible for the separation based on the loadings presented in C&D, (C) PC-1 loading, (D) PC-5 loading

The normal breast spectra presented higher contributions of aromatic CH stretching modes (≈3061 cm^-1^) and CH_2_ asymmetric stretching of lipids and fatty acids ( ≈2946 cm^1^) as presented in Figure 6(Fig. 6). The dominance of lipids in normal tissue has been previously reported when comparing normal and cancerous tissue (Frank et al., 1994[10]; Marro et al., 2014[22]; Talari et al., 2015[34]). The ≈2961 cm^-1^ band caused by Out-of-plane chain end antisymmetric CH_3_ stretch band in lipids and fatty acids vibrations are responsible for this band (Talari et al., 2015[33]) also showed differences in the normal breast and cancer spectra. 

Protein vibrations such as ≈2928, 2881, and 2849 cm^-1^ produced by the symmetric CH_3_ stretch primarily due to protein, CH_2_ asymmetric stretch of lipids and proteins, and symmetric stretch of C-H respectively presented greater contributions on the cancerous spectra. These protein contributions can be attributed to; uncontrolled and abnormal cell proliferation, cell division, and migration in the malignant tissue (Abramczyk and Brozek-Pluska, 2013[1]; Allred et al., 1993[3]). 

#### Hydroxyproline/proline (960-810 cm^-1^)

**Figure 7:**
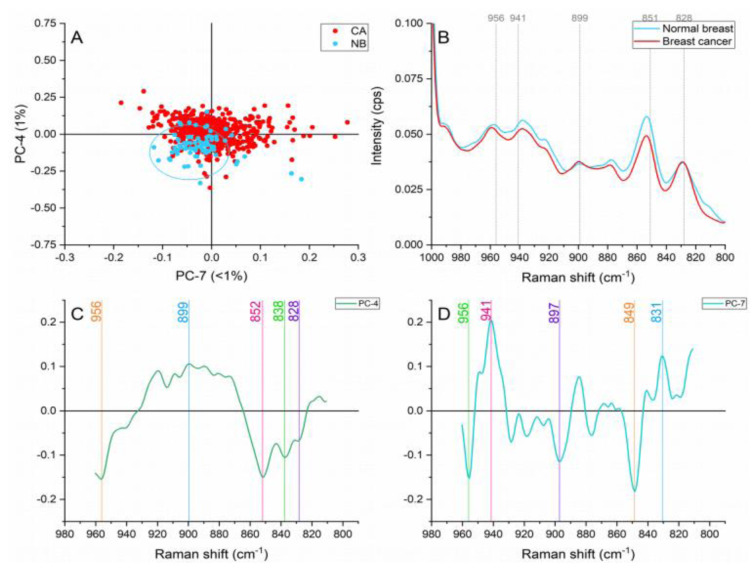
Principal component analysis on the hydroxyproline and proline region (960-810 cm^-1^). (A) Score plot using PC-4 and PC-7 accounting for 2 % of the variance-ellipses were drawn subjectively based on visual trends, (B) Average Raman spectral profile for breast cancer and normal breast. Dotted lines represent the peaks found as responsible for the separation based on the loadings presented in C&D, (C) PC-4 loading, (D) PC-7 loading

Normal breast samples showed increased intensities in the bands located at ≈956 and 851 cm^-1^ in Figure 7(Fig. 7). The former band is representative of carotenoids (Talari et al., 2015[33]). Oleic acid and carotenoids have been identified to dominate normal breast tissue (Zhang et al., 1997[42]) and have demonstrated to play key roles in cellular processes that prevent the development of pathologies in the breast tissue (Abramczyk et al., 2012[2]). The ≈851 cm^-1 ^peak is produced by proline, hydroxyproline and glycogen vibration characteristics of collagen, and which offer structural integrity and biochemical properties to the connective tissue in this case stoma (Martinez et al., 2019[23]).

Within this region, the cancer samples presented greater contributions to the spectral peaks found at ≈941, 899, and 828 cm^-1^. The stretching of C-C skeletal backbone of collagen assigned to the ≈941 cm^-1^ band suggests an adaptation in the ECM on the cancerous samples (Kumar et al., 2013[17]). Structural rearrangements in collagen, resulting in the exposure of proline and hydroxyproline amino acids have shown to affect the intensities of this band (Martinez et al., 2019[23]). The ≈828 cm^-1^ band representing tyrosine vibrations and stretching of PO_2_^-^ in nucleic acids in combination with the adenine peak placed at ≈899 cm^1^ act as an evidence of higher metabolic activity and cell density in the cancerous samples.

## Conclusions

In this study, Raman spectroscopy proved to be a powerful technique for the analysis of *ex vivo* breast tissue. The combination of spectroscopy and a heterogeneous group of TMA breast samples resulted in the successful differentiation of normal breast and breast cancer with a sensitivity of 90 % and specificity of 78 %. DCIS and invasion were both included for analysis, impacting on the heterogeneity of the cohort. 

Dominance of lipids, specifically fatty acids, in the normal tissue is also evident from the results, whereas, protein dominated the malignant breast. The normal breast tissue presented greater contributions of carotenoids, β-carotenoids, and cholesterol which has been reduced in breast cancer cases likely due to lipid peroxidation. Higher protein and nucleic acid contributions were identified in the cancerous samples. Furthermore, greater amide I β-sheet conformations were present in the malignant samples. Results obtained by Raman spectroscopy in this study are comparable those obtained using FTIR on the same cohort of samples reported previously (Lazaro-Pacheco et al., 2020[19]), in which structural changes from α-helix in the normal tissue to β-structures in breast cancer were identified. Ceramide related peaks characterized the breast cancer spectra. The ceramide band could serve as a breast cancer biomarker due to its participation as a structural component of sphingolipids, which are linked to various cellular processes such as cancer development, progression, metastasis and resistance to therapy. 

This model allowed to identify significant biochemical differences associated with the presence of breast cancer. The application of this technology for the study and assessment of breast tissue could facilitate screening of a large number of samples and thus prioritize likely cancerous cases for full examination by the pathologists and alleviate the work within pathology departments. In addition, further work into the atypical and precancerous breast lesions will help identify the earliest chemical signs of onset of malignancy enabling timely prophylactic/interventional strategies. Furthermore, this technology could also be used to assess the early effects of reponse to neoadjuvant therapies at the tissue level; an area of clinical need in breast cancer management. 

## Acknowledgements

DLP doctoral studies were supported by the Mexican Consejo Nacional de Ciencia y Tecnología (CONACYT; CVU 610378), the Secretaría de Educación Pública (Beca complemento 2017-2018 y 2018-2019), and the Mexican Government. AMS is supported by the Birmingham CRUK Centre (C17422/ A25154). We thank Gouri Baldin for the technical assistance in cutting sample blocks.

## Figures and Tables

**Table 1:**
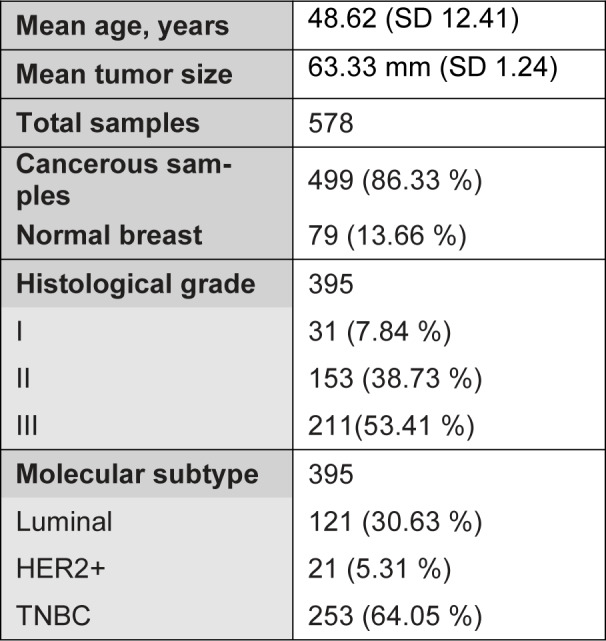
Clinicopathological features of cancerous and normal tissue cohort. Values expressed as n (%), unless otherwise indicated
